# Exposure to oral contraceptives alters human endometrial stem cells culture media secretome

**DOI:** 10.5935/1518-0557.20250194

**Published:** 2026

**Authors:** Raquel Cellin Rochetti, Fernanda Bertuccez Cordeiro, Larissa Berioni Rodrigues da Silveira, Lívia do Vale Teixeira da Costa, Renato Fraietta, Kayla Jane Perkel, Fernando Prado Ferreira, Edson Guimaraes Lo Turco

**Affiliations:** 1 Department of Surgery, Division of Urology, Human Reproduction Section, São Paulo Federal University, São Paulo, Brazil, CEP: 04039-060; 2 Department of Gynecology, São Paulo Federal University, São Paulo, Brazil, CEP: 04039-060; 3 Department of Biomedical Sciences, University of Guelph, Guelph, Ontario, Canada, N1G 2W1; 4 Laboratorio para Investigaciones Biomédicas, Facultad de Ciencias de la Vida, Escuela Superior Politécnica del Litoral, Guayaquil, Ecuador, 090902

**Keywords:** endometrium, cell culture, contraception, stem cell, secretome, metabolites

## Abstract

**Objective:**

To evaluate the effect of oral contraceptives on the secretome of endometrial mesenchymal stem cells (EnMSC) and their potential impact on endometrial plasticity.

**Methods:**

The EnMSC were collected from menstrual shedding of five volunteers and cultured for three passages. Cells were characterized by flow cytometry and culture media was collected at the end of each passage for further secretome analysis. Quantitative analysis included detection of aminoacids, biogenic amines, acylcarnitines, lysophosphatidylcholines, phosphatidylcholines, sphingomyelins, and hexose. Data was analyzed by partial least square analysis. Potential biomarkers were analyzed by receiver operating characteristic (ROC) curve.

**Results:**

From 186 metabolites quantified in the culture media of OC and non-OC groups, 15 metabolites were identified as of high discrimination between groups by the PLS-DA. The ROC curve showed that 4 out of 15 metabolites presented more than 80% of sensitivity. These metabolites are Alanine, Phosphatidylcholine (PC) aa C30:0, Glycine and PC aa C32:2, whose concentrations were higher in the OC group than in non-OC group. The Students’ T-Test analysis confirmed that Alanine was significantly higher in the OC group (*p*=0.00176).

**Conclusions:**

The use of OCs could affect endometrial plasticity and influence the reproductive success. This study provides a preliminary insight into the EnMSCs response to OCs based on specific metabolite signatures, which may contribute to the comprehension of mechanism associated with EnMSC and OCs.

## INTRODUCTION

generation of the human endometrium corresponds to a process in which the tissue thickness increases from 2-4 mm, in the proliferative phase, to 10-15 mm, in the secretory phase (Schwab & Gargett, 2007; Spitzer *et al*., 2012). In a non-conception cycle, tissue breakdown and consequent menstruation occur (Khatun *et al*., 2017). After, the endometrium is repopulated and increases in thickness. Endometrial plasticity and tissue repair are observed after both parturition and miscarriage, events that require tissue regeneration and remodeling (Gellersen & Brosens, 2014).

The reconstitution of the endometrial tissue relies on the presence of endometrial mesenchymal stem cells (EnMSC), which reside in the perivascular space in the endometrium (Gargett & Ye, 2012). Additionally, the main characteristics of EnMSCs are their self-renewal properties and high proliferative potential (Gargett *et al*., 2009). In a cycle regulated by oral contraceptives (OCs), the endometrial changes depend on the type of the hormone, its potency, dosage, and the individual’s health status. Low-dose OCs will stop the proliferation of endometrial glands, affecting the secretory potential of the glands after long-term use (Deligdisch, 1993). The OCs with higher concentrations of progesterone can have more profound effects, such as causing hyperplasia of endometrial vessels and stroma atrophy of the glands (Deligdisch, 1993).

The metabolomics of culture media of EnMSCs may provide new insights into molecular events that may occur in these cells under the effect of OCs. Recent developments in the field of metabolomics-based mass spectrometry (MS) allow for both qualitative and quantitative analysis of metabolic signatures for a given condition, which includes either a single metabolite or a panel of endogenous metabolites (Dias & Koal, 2016). As a result, the secretome of MSC can evidence its influence on various biological processes, including tissue maintenance and regeneration (Praveen Kumar *et al*., 2019).

In this study, we present a quantitative metabolomics analysis of EnMSCs culture media from women taking oral contraceptives. This work aimed to evaluate the effects of exogenous E_2_ and P_4_ on the development and metabolism of EnMSCs by assessing their interaction within a controlled environment. We hypothesize that exposure to exogenous estrogen and progesterone, as present in oral contraceptives, alters the metabolic activity of endometrial mesenchymal stem cells, leading to specific changes in secreted metabolites that may influence endometrial receptivity. Understanding these hormone-driven metabolic shifts could provide novel insights into how OCs modulate endometrial function and help inform future therapeutic strategies to support reproductive health.

## MATERIALS AND METHODS

This was a prospective study carried out at São Paulo Federal University, Brazil. The study received approval from Ethics in Research Committee from the São Paulo Federal University under protocol number 0027/2018. Written informed consent was obtained from all participants.

### Cell isolation and culture

For this study, five women of reproductive age (19 to 29 years old) with normal body mass index (BMI) and regular menstrual cycles (28±1.2 days) were included in two groups according to the use of OC (Low-dose: 0.02 mg): OC group (n=2) and non-OC group (n=3). The endometrial tissue was collected in a non-invasive manner by the disposable menstrual cup (Prudence^®^, São Paulo, Brazil) on the second day of the menstrual cycle. The collector was kept in the vagina for 4 hours and the collected samples were immediately transferred to a falcon tube containing phosphate-buffered saline (PBS - Gibco^®^, Gaithersburg, MD, USA), supplemented with 3% (v/v) Antibiotic-Antimycotic 100x (AA - Gibco^®^, Gaithersburg, MD, USA). Hormone concentration in OCs was not defined for the study. Participants indicated if they were using oral contraceptives that were prescribed by their respective gynecologists. As exclusion criteria, participants that were under other hormonal contraceptive methods; in gynecological treatment; or under assisted reproductive cycles were not included.

The tissue was centrifuged at 400 × *g* for 5 minutes and the supernatant was discarded. The pellet was seeded in a culture flask with a culture medium (Iscove’s Modified Dulbecco’s Medium [IMDM - Gibco^®^, Gaithersburg, MD, USA] supplemented with 10% (v/v) fetal bovine serum and 1% (v/v) AA). The EnMSC that adhered to the plate were cultured in the incubator at 37 ºC and 5% CO_2_, and the culture medium was replaced every 48 h until confluence. The EnMSC were grown up to the second passage (P2) and at the end of each passage (P0, P1, and P2) the culture medium was completely replaced 24h before further culture medium collection for metabolomic analysis, totaling 3 biological replicates for each tissue collected. The culture medium from each passage was centrifuged at 400 × g for 5 minutes, filtered with a 0,2 µm filter, and stored at -80 ºC until metabolomic analysis.

### Cell characterization

The EnMSCs were expanded to the third passage (P3) and characterized by flow cytometry (BD Accuri™ C6, New Jersey, USA) using CD90 and CD105 as positive markers and CD31 and CD45 as negative markers (eBioscience®, Thermo Fisher, Waltham, MA, USA). The analyses were performed by BD Accuri™ C6 software (Becton, Dickinson and Company, New Jersey, USA).

### Quantitative Metabolomics of EnMSC culture medium

Quantitative metabolomics from EnMSC culture media was performed using the AbsoluteIDQ p180 kit (Biocrates Life Sciences AG, Innsbruck, Austria). The amount of 10 µL of culture medium collected after P0, P1, and P2 was used for metabolomic analysis by multiple reaction monitoring methods with electrospray ionization. One biological replicate and one technical replicate was included per group. For this analysis, it was used a API 4000 triple quadrupole mass spectrometer (ABSciex) equipped with an Agilent 1200 Series HPLC and controlled by the software Analyst 1.5 to simultaneous quantify 188 metabolites. This method allowed for the quantification of 188 metabolites, including 21 amino acids and 21 biogenic amines that were initially analyzed based on phenylisothiocyanate (PITC) derivatization. Next, the samples were analyzed by liquid chromatography (LC-) tandem mass spectrometry (-MS/MS) with isotope-labeled internal standards. The concentration of 40 acylcarnitines, 14 lysophosphatidylcholines, 76 phosphatidylcholines, 15 sphingomyelins, and hexose was further analyzed via flow injection analysis (FIA-) MS/MS (Feldman *et al*., 2017).

### Statistical Analysis

The metabolomics dataset was analyzed via a multivariate approach using Metaboanalyst (http://www.metaboanalyst.ca). Multivariate statistics were performed by partial least square-discriminant analysis (PLS-DA) for sample classification and further identification of potential markers for each group. The biomarkers proposed by PLS-DA were analyzed regarding distribution and variability in their respective groups by box plot charts.

The receiver operating characteristic (ROC) curve analysis was performed for the PLS-DA selected variables as a set to observe the sensitivity of the proposed metabolites for correct sample classification in each group (supplementary figure 1). Data was also analyzed by T-test for comparison of metabolites quantification in both Non-OC and OC groups (Supplementary table 1). In addition to the analysis, the original data used for the manuscript was uploaded to the metabolomics workbench under data track ID:1300.

**Table 1:** Univariate statistics performed by T-test shows Means and Standard Deviation (SD) of metabolites detected by quantitative metabolomics.

Metabolite	NO-OCP	OCP	p-value	NO-OCP/OCP
	Mean (SD)			
Ala	-0.621 (0.145)	0.414 (1.120)	**0.00176 (W)**	Down
Arg	-0.041 (0.829)	0.027 (1.148)	0.90191	Down
Asn	0.377 (0.219)	-0.251 (1.242)	0.17356	Up
Asp	-0.203 (0.675)	0.136 (1.189)	0.17378 (W)	Down
Cit	-0.345 (0.809)	0.230 (1.092)	0.29104	Down
Gln	0.377 (0.552)	-0.251 (1.175)	0.24689	Up
Glu	-0.373 (0.664)	0.249 (1.140)	0.25208	Down
Gly	-0.506 (0.431)	0.338 (1.146)	0.07004	Down
His	-0.167 (0.853)	0.111 (1.123)	0.61685	Down
Ile	-0.130 (1.094)	0.087 (0.990)	0.69682	Down
Leu	-0.063 (1.061)	0.042 (1.021)	0.85017	Down
Lys	-0.209 (0.507)	0.139 (1.239)	0.52852	Down
Met	0.049 (0.935)	-0.033 (1.096)	0.88312	Up
Orn	-0.203 (0.695)	0.136 (1.182)	0.54032	Down
Phe	-0.172 (0.860)	0.115 (1.118)	0.6042	Down
Pro	-0.396 (0.198)	0.264 (1.237)	0.152	Down
Ser	0.431 (0.776)	-0.287 (1.068)	0.18215	Up
Thr	-0.298 (0.738)	0.199 (1.140)	0.3655	Down
Trp	0.121 (0.763)	-0.080 (1.170)	0.71763	Up
Tyr	-0.078 (0.734)	0.052 (1.186)	0.81594	Down
Val	-0.173 (0.929)	0.115 (1.083)	0.60268	Down
ADMA	-0.398 (1.171)	0.266 (0.833)	0.21973	Down
alpha-AAA	0.421 (1.534)	-0.281 (0.242)	0.31531	Up
c4-OH-Pro	-0.583 (0.785)	0.389 (0.969)	0.08343 (W)	Down
Carnosine	0.272 (0.618)	-0.181 (1.191)	0.40969	Up
Creatinine	0.128 (0.548)	-0.085 (1.242)	0.76788 (W)	Up
Kynurenine	-0.238 (0.265)	0.158 (1.279)	0.95465 (W)	Down
Met-SO	-0.320 (1.029)	0.214 (0.980)	0.32905	Down
Putrescine	-0.020 (0.810)	0.013 (1.158)	0.76394 (W)	Down
Sarcosine	0.137 (0.321)	-0.091 (1.289)	0.47911 (W)	Up
SDMA	-0.197 (0.633)	0.131 (1.205)	0.55294	Down
Serotonin	0.173 (1.200)	-0.115 (0.901)	0.95465 (W)	Up
t4-OH-Pro	-0.262 (0.359)	0.174 (1.259)	0.34989	Down
Taurine	0.159 (0.445)	-0.106 (1.263)	1 (W)	Up
C0	-0.146 (1.007)	0.098 (1.044)	0.66028	Down
C2	-0.387 (0.723)	0.258 (1.112)	0.23391	Down
C3	-0.152 (0.783)	0.101 (1.157)	0.6491	Down
C4	-0.345 (0.406)	0.230 (1.224)	0.2198	Down
C41	-0.113 (1.048)	0.075 (1.024)	0.84193 (W)	Down
C5	-0.142 (0.422)	0.095 (1.270)	0.61445	Down
C5-OH C3-DC-M	-0.376 (0.860)	0.251 (1.054)	0.24824	Down
lysoPC a C160	-0.060 (0.337)	0.040 (1.294)	0.83109	Down
lysoPC a C161	0.032 (0.404)	-0.021 (1.283)	0.52867 (W)	Up
lysoPC a C170	0.025 (0.308)	-0.017 (1.300)	0.92892	Up
lysoPC a C180	-0.220 (0.358)	0.147 (1.269)	0.43153	Down
lysoPC a C181	0.052 (0.188)	-0.035 (1.313)	0.84998	Up
lysoPC a C182	-0.012 (0.384)	0.008 (1.288)	0.96627	Down
lysoPC a C203	-0.194 (0.901)	0.129 (1.094)	0.64169 (W)	Down
lysoPC a C204	-0.226 (0.223)	0.151 (1.287)	0.41341	Down
lysoPC a C260	-0.003 (0.905)	0.002 (1.113)	0.9931	Down
PC aa C281	-0.413 (0.929)	0.276 (0.998)	0.34535 (W)	Down
PC aa C300	-0.607 (0.664)	0.405 (1.007)	0.05048	Down
PC aa C302	-0.349 (0.894)	0.233 (1.048)	0.28592	Down
PC aa C320	0.024 (0.572)	-0.016 (1.243)	0.94339	Up
PC aa C321	-0.305 (1.057)	0.203 (0.967)	0.38841 (W)	Down
PC aa C322	-0.560 (0.946)	0.373 (0.894)	0.05912 (W)	Down
PC aa C323	-0.046 (1.288)	0.031 (0.843)	0.59555 (W)	Down
PC aa C341	0.126 (0.718)	-0.084 (1.187)	0.70526	Up
PC aa C342	-0.266 (0.788)	0.177 (1.128)	0.42094	Down
PC aa C343	-0.087 (0.898)	0.058 (1.112)	0.86394 (W)	Down
PC aa C344	0.528 (0.952)	-0.352 (0.914)	0.09551	Up
PC aa C361	0.098 (0.500)	-0.065 (1.258)	0.60699 (W)	Up
PC aa C362	0.070 (0.574)	-0.047 (1.240)	0.67836 (W)	Up
PC aa C363	-0.086 (0.378)	0.057 (1.285)	0.76068	Down
PC aa C364	0.183 (0.682)	-0.122 (1.190)	0.58197	Up
PC aa C365	0.429 (0.792)	-0.286 (1.062)	0.18398	Up
PC aa C366	0.559 (0.981)	-0.372 (0.871)	0.07552	Up
PC aa C380	0.437 (0.522)	-0.291 (1.158)	0.17543	Up
PC aa C381	0.265 (0.966)	-0.176 (1.039)	0.42318	Up
PC aa C383	0.252 (0.623)	-0.168 (1.195)	0.77562 (W)	Up
PC aa C384	0.208 (0.552)	-0.139 (1.227)	0.53069	Up
PC aa C385	0.353 (0.510)	-0.236 (1.196)	0.27958	Up
PC aa C386	0.370 (0.508)	-0.246 (1.191)	0.25709	Up
PC aa C402	-0.414 (1.006)	0.276 (0.950)	0.20096	Down
PC aa C403	0.356 (0.920)	-0.237 (1.031)	0.27637	Up
PC aa C404	-0.101 (0.889)	0.067 (1.115)	0.38841 (W)	Down
PC aa C405	0.270 (0.341)	-0.180 (1.260)	0.33427	Up
PC aa C406	0.394 (0.492)	-0.262 (1.185)	0.22558	Up
PC aa C421	0.397 (0.741)	-0.265 (1.100)	0.22117	Up
PC aa C424	-0.014 (0.385)	0.009 (1.287)	0.96068	Down
PC aa C425	0.227 (0.861)	-0.151 (1.105)	0.49334	Up
PC ae C321	-0.450 (0.877)	0.300 (1.008)	0.16232	Down
PC ae C322	-0.547 (0.357)	0.365 (1.138)	0.04823	Down
PC ae C340	0.180 (0.423)	-0.120 (1.264)	0.52393	Up
PC ae C341	0.009 (0.680)	-0.006 (1.209)	0.97855	Up
PC ae C342	-0.042 (0.984)	0.028 (1.069)	0.89906	Down
PC ae C343	-0.262 (0.780)	0.175 (1.133)	0.42728	Down
PC ae C361	0.115 (0.806)	-0.077 (1.152)	0.77562 (W)	Up
PC ae C362	0.184 (0.620)	-0.123 (1.211)	0.5792	Up
PC ae C363	0.277 (0.604)	-0.185 (1.194)	0.40139	Up
PC ae C364	-0.075 (1.183)	0.050 (0.931)	0.38841 (W)	Down
PC ae C365	0.281 (0.522)	-0.187 (1.217)	0.39401	Up
PC ae C381	0.143 (0.561)	-0.096 (1.236)	0.9061 (W)	Up
PC ae C382	0.320 (0.561)	-0.213 (1.194)	0.33021	Up
PC ae C383	0.288 (0.728)	-0.192 (1.147)	0.38175	Up
PC ae C384	0.139 (0.551)	-0.093 (1.239)	0.67649	Up
PC ae C385	-0.024 (0.956)	0.016 (1.085)	0.9437	Down
PC ae C386	-0.016 (0.445)	0.011 (1.275)	0.95373	Down
PC ae C401	0.222 (0.928)	-0.148 (1.072)	0.50217	Up
PC ae C402	-0.544 (0.644)	0.363 (1.059)	0.08464	Down
PC ae C403	-0.185 (0.611)	0.123 (1.214)	0.57874	Down
PC ae C404	-0.010 (0.881)	0.007 (1.124)	0.97505	Down
PC ae C405	0.262 (0.560)	-0.175 (1.212)	0.42731	Up
PC ae C406	0.246 (0.570)	-0.164 (1.213)	0.45652	Up
PC ae C422	0.277 (1.245)	-0.185 (0.827)	0.4005	Up
PC ae C423	-0.287 (0.981)	0.192 (1.022)	0.38307	Down
SM OH C141	0.188 (0.682)	-0.126 (1.189)	0.57078	Up
SM OH C161	0.459 (0.806)	-0.306 (1.040)	0.1534	Up
SM OH C221	-0.168 (0.664)	0.112 (1.200)	0.61382	Down
SM OH C222	-0.018 (0.659)	0.012 (1.216)	0.9559	Down
SM OH C241	-0.185 (0.719)	0.123 (1.176)	0.57757	Down
SM C160	0.089 (0.600)	-0.059 (1.231)	0.79057	Up
SM C161	0.122 (0.855)	-0.081 (1.129)	0.95465 (W)	Up
SM C180	0.071 (0.610)	-0.047 (1.229)	0.8324	Up
SM C181	0.023 (0.690)	-0.015 (1.205)	0.94509	Up
SM C202	-0.213 (1.006)	0.142 (1.030)	0.59256 (W)	Down
SM C240	0.044 (0.544)	-0.029 (1.250)	0.89484	Up
SM C241	-0.090 (0.560)	0.060 (1.242)	0.78668	Down
SM C260	0.437 (1.031)	-0.291 (0.920)	0.17531	Up
SM C261	-0.041 (0.701)	0.027 (1.200)	1 (W)	Down
H1	0.150 (0.983)	-0.100 (1.057)	0.65273	Up

*p*-value is calculated with t-test as a default.

*p*-value with (W) is calculated by the Wilcoxon Mann Whitney test

**Figure 1:**
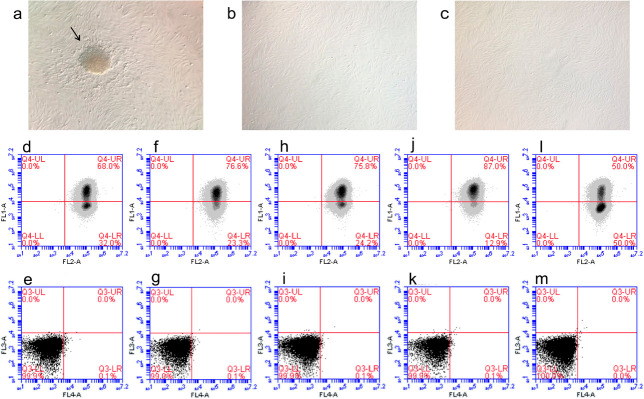
Characterization of EnMSC cultured *in vitro*. a-c: Morphology observed by a phase contrast microscope in 4x objective. a: Cells in passage 0 evidencing colony formation indicated by the black arrow. b and c: Cells in passage 1 and 2 respectively, without colony. d-m: Flow cytometry analysis with positive markers CD90 (FL-1) and CD105 (FL-2) and negative markers CD31 (FL-3) and CD45 (FL-4) of each volunteer. d-i: OC group. J-m: Non-OC group.

The quantification data analyzed by Analyst 1.5 underwent univariate T-test statistics to identify significant metabolites between groups and Wilcoxon Mann Whitney test. *P* value was set as 0.005 for significance.

## RESULTS

The evaluation of cell morphology by a phase contrast microscope (Olympus IX71 - Olympus Corporation, Tokyo, Japan) with a coupled digital camera showed a colony formation in P0, but this morphology was absent in the following passages (Figure 1, A-C). The isolated cells were characterized as mesenchymal stem cells in P3 with a range of 50% to 87% of positive markers (CD90 and CD105) and 0% to 0.1% of negative markers (CD31 and CD45) (Figure 1, D_1_-H_2_), confirming cells characterization and providing further proof that loss of colony formation does not associate with differentiation into other cell types.

**Figure 2:**
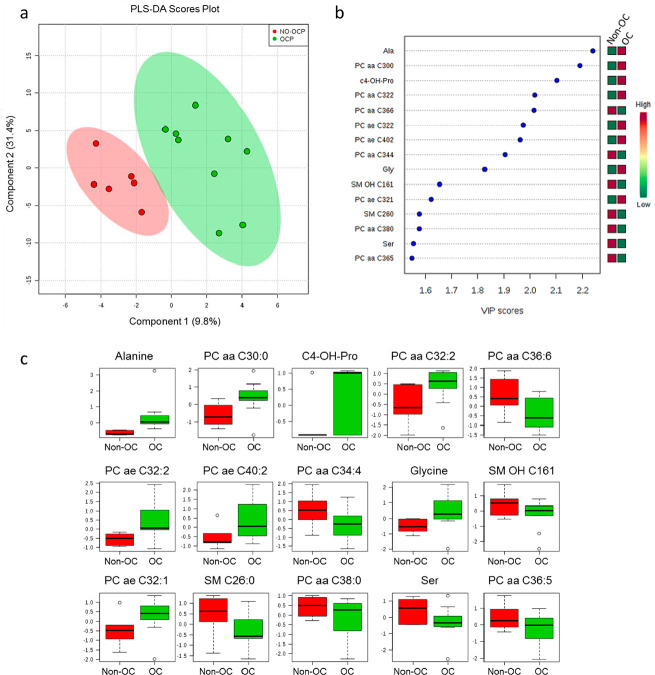
a- PLS-DA Scores Plot demonstrates groups separation considering the metabolomic quantitative analysis of culture media from women using OC (green circles) and not using OC (red circles). b- VIP scores considering the variables of most importance for the PLS-DA regression model. Red boxes indicate metabolites oh higher concentration of the respective metabolite (indicated by abbreviation) for the corresponding group, whereas green boxes indicate lower concentrations of the metabolites for the corresponding groups (Non-OC or OC groups). c- Box plot charts indicate variabilities of samples distribution, and it is possible to observe differences in the metabolites concentration for each group of the study.

The quantitative metabolomics approach was analyzed by multivariate statistics to observe characteristics of the culture medium of EnMSC. The PLS-DA regression was performed for predicting sample classification based on the use of OCs, and a complete group separation was observed (Figure 2A). Individual molecules identified as biomarkers by PLS-DA had their VIP Scores shown in Figure 2B.

In addition, the potential biomarkers were individually analyzed for their variability and distribution by the box plot charts, which also showed that 8 out of 15 molecules were significantly increased in women using OCs, whereas 7 biomarkers demonstrated higher concentrations in the non-OC group (Figure 2C).

Our data revealed higher concentration of alanine, hydroxyproline, glycine, and phosphatidylcholine in the OC group, whereas serine, sphingomyelin, and phosphatidylcholine metabolites were more concentrated in culture media from EnMSC of women not using OC.

**Figure 3:**
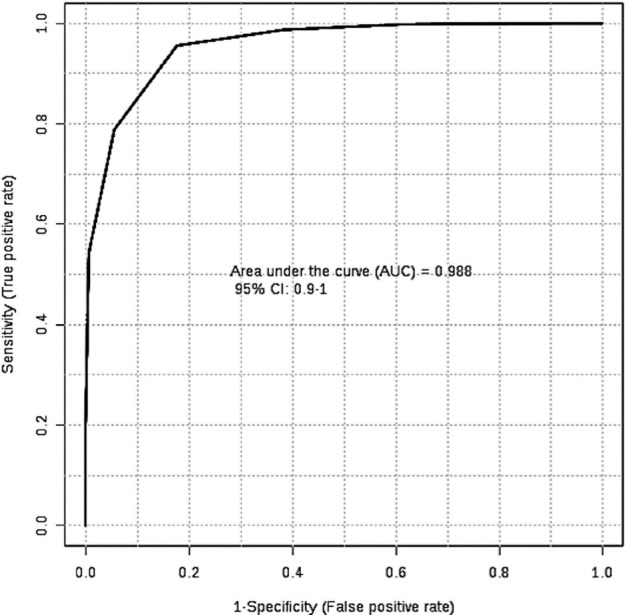
ROC curve analysis shows an AUC= 0.988, considering a confidence interval of 95%. The x axis indicates the Specificity whereas the y axis indicates the Sensitivity of the analysis, by including the set of biomarkers proposed by the PLS-DA regression model.

This result may be a consequence of being on OCs for 5 years or more. To assure sensitivity and specificity of proposed biomarkers, the ROC curve analysis was performed considering the PLS-DA selected biomarkers as a set. The ROC curve demonstrated an area under the curve (AUC) of 0.988 (Figure 3) and 100% of correct classification for samples as OC or non-OC.

## DISCUSSION

The EnMSCs role in endometrium repopulation is orchestrated by altered stimulus from E_2_ and P_4_ that induces decidualization (Maruyama *et al*., 2010). Recently, the study of these cells has been in the spotlight given the range of associations with various diseases, such as ovarian cancer, and infertility associated with recurrent implantation failure (Bu *et al*., 2016; Peter Durairaj *et al*., 2017). Moreover, EnMSC is easily accessible and presents relevant immunomodulatory properties (Khatun *et al*., 2017).

In culture, the EnMSC are characterized by their adherent and colony-forming properties. Furthermore, they possess distinct markers that are readily characterized via flow cytometry (Du *et al*., 2016). Using metabolomics, recent studies have been focusing on the secretome of cells in culture, aiming to better understand how cells behave under certain environments (Peter Durairaj *et al*., 2017). For the present study, the evaluation of culture medium provides a first insight into the role of EnMSC in women taking OCs. This novel information has contributed to improving our knowledge concerning endometrial response to exogenous hormones at the metabolic level. Clinically, the long-term use of combined OC (5 years or more) has been shown to influence the production of endometrial cells by decreasing endometrial thickness (Talukdar *et al*., 2012).

These reports support the differences found in the EnMSCs metabolic response to OC in the present study, by visualization of clear separation between groups in the PLS-DA (Figure 2a). The box plot charts of identified metabolites as indicated by the PLS-DA demonstrated that the differences found are consistent between groups.

According with potential molecular mechanism, we found the following biological pathways:

### 1. Immunomodulation and Inflammation

Alanine, which was significantly increased in the OC group (*p*=0.00176, Table 1), may reflect immunomodulatory activity. While the role of OCs in alanine metabolism is not well described, previous work suggests that EnMSC can protect against hepatic inflammation, modulating markers such as serum alanine aminotransferase and pro-inflammatory cytokines (Lu *et al*., 2016). Although enzymatic activity was not measured here, the increased alanine in the OC group may be linked to hormonal effects (Chen & Kotani, 2012).

### 2. Energy Metabolism and Amino Acid Utilization

Serine was more abundant in the non-OC group and considered a potential marker based on PLS-DA results (Figure 2b), although without statistical difference in T-test (*p*=0.18215). Previous studies have shown that estrogen can enhance the mTOR pathway, which metabolizes serine to support cell proliferation (Hsu *et al*., 2016). In the context of endometrial cancer, such regulation has been linked to hormone-driven growth (Zhu *et al*., 2016). The lower serine levels in the OC group may reflect increased metabolic use driven by exogenous hormones.

### 3. Membrane Lipid Metabolism

Sphingomyelin and phosphatidylcholine were more concentrated in the non-OC group. These lipids play roles in cell membrane integrity and signaling. Prior studies associate high-dose OCs with elevated plasma sphingolipids and thrombosis risk (Oyelola *et al*., 1990). However, our findings suggest that low-dose OCs may exert a different, possibly suppressive, effect on these lipid classes in EnMSCs.

### 4. Angiogenesis and Tissue Integrity

Other types of phosphatidylcholine levels were also elevated in the OC group. Although less studied in EnMSC, phosphatidylcholines are precursors for signaling molecules involved in angiogenesis and tissue integrity. The altered profile observed here could be part of the long-term hormonal modulation of endometrial microvasculature, consistent with prior findings showing endometrial thinning after prolonged OC use (Talukdar *et al*., 2012).

Therefore, our study presented metabolite alterations in culture media from EnMSC that may be a result of altered metabolism of these cells under OCs effects. The use of OCs could affect endometrial characteristics that are crucial for reproductive success, such as endometrial receptivity. Importantly, obstetric outcomes could not be evaluated in this study, as all participants were not attempting pregnancy. Instead, we focused on the secretome profile of endometrial stem cells to explore potential effects of OCs in EnMSCs physiology.

This study includes preliminary data, and further studies must be conducted to validate differences found and to investigate underlying mechanism associated with EnMSC and OCs. This study provides a preliminary insight into the EnMSCs differential biological response to OCs based on specific metabolite signatures, which may contribute to the development of future new therapies.
